# 2OMe-LM: predicting 2′-O-methylation sites in human RNA using a pre-trained RNA language model

**DOI:** 10.1093/bioinformatics/btaf417

**Published:** 2025-07-29

**Authors:** Qianpei Liu, Min Zeng, Yiming Li, Chengqian Lu, Shichao Kan, Fei Guo, Min Li

**Affiliations:** School of Computer Science and Engineering, Central South University, Changsha 410083, China; School of Computer Science and Engineering, Central South University, Changsha 410083, China; School of Computer Science and Engineering, Central South University, Changsha 410083, China; School of Computer Science, Key Laboratory of Intelligent Computing and Information Processing, Xiangtan University, Xiangtan, Hunan 411105, China; School of Computer Science and Engineering, Central South University, Changsha 410083, China; School of Computer Science and Engineering, Central South University, Changsha 410083, China; School of Computer Science and Engineering, Central South University, Changsha 410083, China

## Abstract

**Motivation:**

2′-O-methylation (2OMe) is a common post-transcriptional modification in RNA that plays a crucial role in regulating gene expression and is implicated in various biological processes and diseases. Computational methods offer an efficient alternative to the time-consuming and costly experimental identification of 2OMe sites. Recent advancements in RNA pre-trained language models have revolutionized RNA bioinformatics. However, there remains a gap in their application specifically for predicting 2OMe sites.

**Results:**

In the study, we propose a novel deep learning framework, 2OMe-LM, for predicting 2OMe sites in RNA. 2OMe-LM integrates RNA sequence features derived from RNA pre-trained language models with those obtained from the word2vec technique. Then, 2OMe-LM employs fully connected layers and a bidirectional long short-term memory network to process the two types of features separately, followed by a feature fusion module for the final prediction. Additionally, an attention block is incorporated to provide the interpretability of the prediction results. The results demonstrate that 2OMe-LM significantly outperforms existing state-of-the-art predictors, with features from RNA pre-trained language models proving to be critical. Motif analysis further demonstrates 2OMe-LM’s potential for discovering 2OMe-related motifs.

**Availability and implementation:**

The 2OMe-LM web server is available at https://csuligroup.com:9200/2OMe-LM. The source code can be obtained from https://github.com/CSUBioGroup/2OMe-LM.

## 1 Introduction

Recent advances in RNA epigenetics have identified over 170 distinct types of RNA modifications (Cappannini *et al.* 2024). Among them, 2′-O-methylation (2OMe) occurs when a methyl group is transferred from S-adenosylmethionine to the 2′ hydroxyl group of the ribose sugar in the RNA backbone. This modification is typically catalysed by specific methyltransferases, such as fibrillarin, in the presence of small nucleolar RNAs that serve as guide RNAs for the site-specific methylation process (Paramasivam 2020). 2OMe is a prevalent post-transcriptional modification found in various types of RNA, including miRNA, tRNA and mRNA (Zhao *et al.* 2017). It plays a crucial role in distinguishing between self and non-self RNA molecules, which is essential for immune response mechanisms. Cellular RNAs typically undergo 2OMe modification, marking them as ‘self’ and helping to prevent an immune response against them. In contrast, viral RNAs often lack this modification, categorizing them as ‘non-self’, thereby triggering an immune defense (Picard-Jean *et al.* 2018). Recent studies have shown that 2OMe is closely associated with complex diseases, such as thalassemia (Sornjai *et al.* 2017) and congenital muscular dystrophy (Bosgra *et al.* 2019).

Currently, various experimental methods are available for detecting 2OMe. Classical biochemical methods, such as liquid chromatography coupled with mass spectrometry (Douthwaite and Kirpekar 2007) and two-dimensional thin-layer chromatography (Grosjean *et al.* 2007), can be effective but are time-consuming and labour-intensive. More advanced high-throughput techniques based on deep sequencing, including Nm-seq (Dai *et al.* 2017), RiboMeth-seq (Krogh *et al.* 2017), 2OMe-seq (Incarnato *et al.* 2017), Nm-REP-seq (Zhang *et al.* 2023), Nm-Mut-seq (Chen *et al.* 2023) and RibOxi-seq (Zhu *et al.* 2017), offer satisfactory performance but still costly, time-consuming and require specialized expertise. This highlights the importance of developing computational methods to complement experimental approaches for predicting 2OMe sites.

Several computational methods have been developed for predicting RNA 2OMe sites. Chen *et al.* (2016) introduced the first computational tool to identify 2OMe sites, utilizing nucleotide chemical properties and composition as features on a benchmark dataset constructed from RMBase (Sun *et al.* 2016) to develop a support vector machine (SVM)-based predictor. Then, Mostavi *et al.* (2018) proposed Deep-2′-O-Me, which employs sequence embeddings and a convolutional neural network (CNN) layer for predicting RNA 2OMe sites. iRNA-2OM (Yang *et al.* 2018) and iRNA-PseKNC (2methyl) (Tahir *et al.* 2019) were developed, using SVM and CNN layers, respectively, while relying on the same dataset as Chen *et al.* (2016). Following the provision of a new reliable dataset by Dai *et al.* (2017) and Hsu et al. (2019) through an improved Nm-seq technique [Gene Expression Omnibus (GEO) Accession: GSE90164], Zhou *et al.* (2018), who had previously developed NmSEER, used this fresh Nm-seq dataset to create training and independent test sets for developing NmSEER V2.0 (Zhou *et al.* 2019). Li *et al.* (2021) leveraging a combination of CNN and bidirectional long short-term memory networks (Bi-LSTM), captured critical features of the full mRNA sequence to construct DeepOMe, a multi-to-multi predictor. Ao *et al.* (2022) developed NmRF, a predictor based on optimal mixed features and a random forest classifier capable of identifying modification sites across multiple species. BERT2OME (Soylu and Sefer 2023), another cross-species predictor for 2OMe sites, transforms each RNA sequence into vector embeddings using a BERT model, which are then analysed with a 2D CNN to learn their features. By integrating experimental data generated from RMBase and Nm-seq, Yang *et al.* (2023) constructed a larger dataset that further classified 2OMe into four types (A, C, G, U) and proposed a two-step feature selection model, i2OM. Pham *et al.* (2024) introduced H2Opred, a novel hybrid deep learning model, while Harun-Or-Roshid *et al.* (2024) presented Meta-2OM through a meta-learning approach.

Although existing computational methods have made significant contributions to the prediction of 2OMe sites, they have certain limitations. Typically, these methods rely traditional encoding techniques for RNA sequences, such as k-mer and one-hot coding, ignoring the rich biological information. Recent advancements in large RNA language models have revolutionized the field of RNA bioinformatics (Ding *et al.* 2022, Hou *et al.* 2025, Zeng *et al.* 2025), enhancing downstream tasks such as RNA structure prediction and RNA interaction prediction. However, a significant gap remains in the application of pre-trained RNA language models specifically for predicting 2OMe sites. These RNA language models are trained on hundreds of millions of natural RNA sequences, and learn multi-dimension biological information that contain structural/functional properties as well as evolutionary information, providing a rich representation for predicting 2OMe sites.

In this study, we propose 2OMe-LM, the first interpretable deep learning framework that leverages a pre-trained RNA language model for predicting 2OMe sites. 2OMe-LM encodes RNA sequences from two distinct perspectives. First, 2OMe-LM incorporates a pre-trained language model, SpliceBERT, to encode RNA sequences, embedding multi-dimension biological information. In addition, 2OMe-LM uses the word2vec technique to encode RNA sequences. The embedding representations derived from SpliceBERT and the word2vec model are processed through fully connected layers and a Bi-LSTM block, respectively. Subsequently, the two types of features are fused and passed into an attention block. 2OMe-LM introduces a multi-head self-attention mechanism, allowing the fused features to obtain attention weights for each nucleotide. Finally, the output from the attention block is passed through several fully connected layers to perform the 2OMe site prediction task.

To evaluate the performance of 2OMe-LM, we compared it with existing state-of-the-art predictors using independent test sets. The results demonstrate that 2OMe-LM outperforms existing predictors across most evaluation metrics. An ablation study and t-SNE visualization highlight the effectiveness of the RNA sequence embeddings encoded by the pre-trained RNA language model. Furthermore, motif analysis indicates that 2OMe-LM can capture relevant sequence motifs, which enhances the interpretability of the model’s predictions. Finally, to facilitate the use of 2OMe-LM, we developed a user-friendly web server.

## 2 Materials and methods

### 2.1 Datasets

In this study, We collected a total of 15 637 entries by integrating 8490 records from RMBase v3.0 (Xuan *et al.* 2018, 2024) with experimentally validated data from several established methodologies, including Nm-seq (GSE90164), Nm-Mut-seq (GSE174518), 2OMe-seq (GSE73065), RiboMeth-seq (GSE77024), RibOxi-seq (GSE96999), and Nm-REP-seq (GSE157930, GSE198748). The dataset construction followed a rigorous multi-step procedure:

1. We aggregated 2OMe site information from multiple sources and extracted 41-nucleotide sequences centred on the modification site, with the 21st nucleotide representing the 2OMe site. All thymine (‘T’) residues were converted to uracil (‘U’).
2. For mRNA transcripts, negative samples were drawn from the same transcript sequences. For other RNA types, negative samples were selected from the same RNA molecules. A 1:2 ration of positive to negative samples was maintained, with 41-nt fragments did not overlap with any known modification sites.
3. To reduce sequence redundancy, we applied CD-HIT-EST (Li and Godzik 2006) with an 80% sequence identity threshold to both positive and negative samples.
4. To create a balanced dataset, non-2OMe (negative) samples were down-sampled, resulting in an equal number of positive and negative samples.

As a result, the final dataset consisted of 8037 positive and 8037 negative samples. These were subsequently partitioned into training and independent test sets using an 8:2 ratio. Table 1, available as supplementary data at *Bioinformatics* online, provides a statistical summary of the dataset.

**Table 1.** Performance comparison of 2OMe-LM and deep learning baseline models using 5-fold CV.^a^

Deep learning baseline models	ACC	F1-score	Precision	Recall	AUC	AUPR	MCC
GloVe + MLP	0.795	0.780	0.843	0.725	0.890	0.872	0.596
GloVe + TextCNN + MLP	0.746	0.718	0.805	0.650	0.842	0.823	0.502
GloVe + Transformer + MLP	0.742	0.720	0.787	0.664	0.832	0.815	0.490
Word2vec + MLP	0.813	0.811	0.819	0.803	0.891	0.906	0.626
Word2vec + TextCNN + MLP	0.764	0.761	0.772	0.751	0.844	0.852	0.529
Word2Vec + Transformer + MLP	0.749	0.741	0.766	0.718	0.828	0.833	0.499
2OMe-LM	**0.846**	**0.841**	**0.873**	**0.811**	**0.919**	**0.929**	**0.695**

a The best performance values are highlighted in bold.

### 2.2 Model architecture

**Figure 1.**
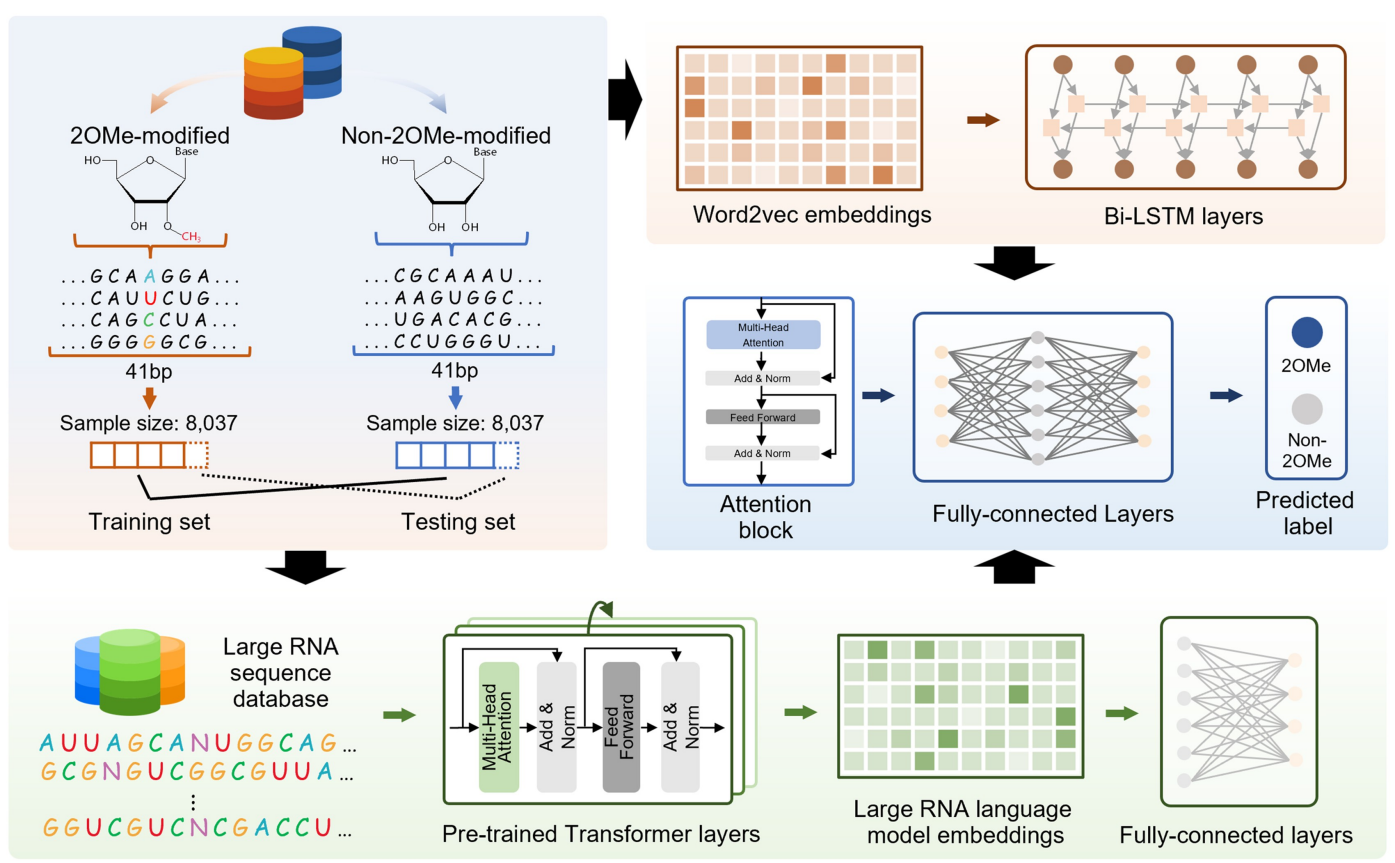
Architecture of 2OMe-LM. 2OMe-LM takes RNA sequences as inputs, which are encoded using both a pre-trained RNA language model and the word2vec technique. The embedding derived from the pre-trained RNA language model is passed through a fully connected layer for dimensionality reduction, while the embedding generated by the word2vec technique is processed by a Bi-LSTM. Subsequently, the two types of embeddings are fused, and the fused features are fed into an attention block. Finally, the output from the attention block is passed through three fully connected layers to perform the 2OMe site prediction task.

As shown in Fig. 1, 2OMe-LM is an end-to-end deep learning model that takes RNA sequences as inputs for the 2OMe site prediction task. Before being processed by 2OMe-LM, the input RNA sequence is encoded using both a pre-trained RNA language model and the word2vec technique, resulting in two types of embeddings for the same RNA sequence. The embedding derived from the pre-trained RNA language model is passed through a fully connected layer for dimensionality reduction, while the embedding generated by the word2vec technique is processed by a Bi-LSTM. Subsequently, the two types of embeddings are fused, and the fused features are fed into an attention block. Finally, the output from the attention block is passed through three fully connected layers to perform the 2OMe site prediction task.

#### 2.2.1 Pretrained RNA language model

In this study, we used a pre-trained RNA language model, SpliceBERT (Chen *et al.* 2024), which is based on the Bidirectional Encoder Representations from Transformers (BERT) architecture. SpliceBERT consists of six Transformer encoder layers, each with 512 hidden units and 16 attention heads, enabling it to process input sequences up to 1024 nucleotides in length and generate embeddings that are valuable for downstream tasks such as splice site prediction and variant effect analysis. The training of SpliceBERT employs masked language modelling, a self-supervised approach in which 15% of the nucleotides in each input sequence are randomly masked. The model learns to predict the correct nucleotide identity at each masked position, effectively capturing intrinsic sequence dependencies and biological motifs without labelled data. This multi-species pre-training approach, applied to more than 2 million RNA sequences from 72 vertebrate species, allows SpliceBERT to capture conserved regulatory signals across vertebrates, making it highly effective for cross-species genomic analyses. By leveraging such diverse training data, SpliceBERT captures evolutionary conservation within primary sequences. We utilized SpliceBERT as an encoding method to represent RNA sequences. It outputs RNA representations with dimensions of (L+2)×512, where 512 is the embedding dimension, *L* is the length of the RNA sequence. The additional 2 corresponds to the special tokens ‘[CLS]’ and ‘[SEP]’ added at the beginning and end of each sequence, respectively. By leveraging the advanced feature representation capabilities of SpliceBERT, 2OMe-LM generates high-quality RNA sequence representations, effectively enhancing the knowledge available to the neural network.

#### 2.2.2 Word2vec embedding

The word2vec (Mikolov *et al.* 2013) technique is a widely used word embedding method that encodes words in text into numerical vector representations. In this study, we applied it to encode RNA sequences, treating them similarly to natural language. RNA sequences can be viewed as a language composed of four types of nucleotides (A, U, C, G), where each nucleotide or its combination (such as base pairs or longer fragments) can be considered as words.

Specifically, we treat a k-mer as a word. For an RNA sequence of length L, this results in L-k+1 k-mers. We trained a word2vec model using the continuous bag of words (CBOW) model, where the model predicts a target k-mer based on the surrounding context k-mers in a specified window. Each k-mer is input into the word2vec model, generating vectors of length d. Consequently, for each RNA sequence, we obtain an embedding of size (L-k+1, d).

#### 2.2.3 Feature fusion and prediction

The RNA sequences are encoded separately using SpliceBERT and the word2vec technique. The features obtained from SpliceBERT are processed through a fully connected layer for dimensionality reduction, while those from word2vec are fed into a Bi-LSTM to capture long-term dependencies in the sequential data. The Bi-LSTM, an extension of the LSTM, combines two LSTM layers operating in opposite directions to extract contextual information from both before and after a given point in the sequence, allowing the model to understand the data more comprehensively. The forward propagation of the LSTM is described as follows:

(1) ft=σ(Wf·[ht−1,xt]+bf)

 

(2) it=σ(Wi·[ht−1,xt]+bi)

 

(3) c∼t=tanh(Wc·[ht−1,xt]+bc)

 

(4) ct=ft∘ct−1+it∘c∼t

 

(5) ot=σ(Wo·[ht−1,xt]+bo)

 

(6) ht=ot∘tanh⁡ct

where $x_{t}$ is the input at time step t, $h_{t}$ represents the hidden state at time step t, b denotes the bias, $f_{t}$, $i_{t}$ and $o_{t}$ represent the outputs of the forget gate, input gate and output gate at time step t, respectively. $c_{t}$ and ${\tilde{c}}_{t}$ denote the cell state and its update at time step t, respectively. By configuring the parameters of the hidden layer, the output dimension of the Bi-LSTM can be aligned with the input dimension.

To integrate the processed features from both encoders, 2OMe-LM applies a linear interpolation method to adjust the second dimension of the two embeddings to a sequence length of L, thereby mapping all features back to the sequence level. Subsequently, the embeddings are concatenated along their third dimension and fed into an attention block. The attention block functions similarly to a Transformer encoder layer (Vaswani *et al.* 2017, Wu *et al.* 2023, Zeng *et al.* 2023), enhancing the utilization of contextual information embedded within these high-dimensional representations. The multi-head self-attention mechanism in the attention block enables the model to capture the relationships between different parts of the RNA sequence embeddings. By processing the embeddings with multiple attention heads, the model enhances its ability to recognize complex biological interactions encoded within the embeddings. Following this, a position-wise feed-forward network transforms the output from the attention mechanism, with each position’s embedding being processed through the feed-forward network. Additionally, the layer incorporates residual connections followed by layer normalization, which stabilizes the learning process and facilitates effective integration of the model’s components. To prevent overfitting, dropout regularization is applied within the layer.

Finally, 2OMe-LM employs fully connected layers to perform 2OMe site prediction. After flattening the fused features, a three-layer fully connected network is used to process the high-dimensional features. The final output of the neural network represents the probability of the presence of a 2OMe site at the centre of the RNA sequence.

### 2.3 Deep learning baseline models

In this study, we aim to develop advanced deep learning frameworks for the accurate prediction of 2OMe sites in RNA sequences. To validate the performance of our proposed model, 2OMe-LM, we conducted comprehensive comparisons against several deep learning baseline models. The comparative models are described as follows:

1. GloVe (Pennington *et al.* 2014) + MLP, this model encodes RNA sequences using the GloVe embedding technique. The resulting sequence representations are then passed through a multilayer perceptron (MLP) to predict 2OMe modification sites.
2. GloVe + TextCNN (Kim 2014) + MLP, this model transforms RNA sequences into vectors using the GloVe embedding technique. A Text Convolutional Neural Network (TextCNN) is then applied to capture local contextual features. These extracted features are subsequently fed into an MLP for classification.
3. GloVe + Transformer + MLP, this model encodes RNA sequences using GloVe embedding technique, and then processes them using a Transformer to capture sequence dependencies. The outputs are passed through an MLP to estimate the probability of modification at each site.
4. Word2vec + MLP, this model uses the Word2Vec embedding method to generate vector representations of RNA sequences, which are then input into an MLP for prediction of 2OMe sites.
5. Word2vec + TextCNN + MLP, this model leverages Word2Vec to embed RNA sequences, followed by a TextCNN to extract local sequence features. These are then passed through an MLP for final classification.
6. Word2Vec + Transformer + MLP, this model utilizes Word2Vec to encode sequences, followed by a Transformer to model sequence dependencies. An MLP layer is used to produce the final prediction outputs.

In the study, we used grid search strategy to find the optimal parameters for these deep learning baseline models.

### 2.4 Evaluation metrics

To evaluate the performance of 2OMe-LM and existing predictors, we used some commonly used evaluation metrics, including accuracy (ACC), F1-score, precision, recall, area under the curve (AUC), area under the precision–recall curve (AUPR) and Matthews correlation coefficient (MCC). These evaluation metrics are calculated using the following formulas:

(7) Acc=TP+TNTP+TN+FP+FN

 

(8) Precision=TPTP+FP

 

(9) Recall=TPTP+FN

 

(10) F1-score=2×Precision×RecallPrecision+Recall

 

(11) MCC=TP×TN-FP×FN(TP + FP)(TP + FN)(TN + FP)(TN + FN)

where TP, TN, FP, and FN represent true positive, true negative, false positive, and false negative, respectively.

### 2.5 Implementation details

2OMe-LM is implemented using PyTorch. We employed the Adam optimizer with a learning rate of 0.0005. To stabilize model training, we adopted a WarmupLR strategy, where the learning rate is linearly increased from 0 to 0.0005 over the course of 150 warm-up steps. In the word2vec module, the k-mer size is set to 4, and the embedding dimension is set to 128. The prediction layer uses a sigmoid activation function to compute the output, which ranges from 0 to 1, representing the predicted probability. During model training, an early stopping mechanism is employed. This mechanism monitors the AUC value on the validation set and stops training if the AUC does not improve for 100 consecutive epochs. The batch size is set to 32 and all dropout rates are set to 0.2. We utilized Binary Cross Entropy Loss (BCELoss) as the loss function. In a binary classification problem with N samples, where each sample $x_{i}$ is associated with a binary label $y_{i}\in \;\{0,\;1\}$, and the model predicts the probability $p_{i}\in [0,1]\;$of the sample belonging to the positive class, the BCELoss is defined as the average of the negative log-likelihood of the predicted probability for each sample:

(12) BCELoss=-1N∑i=1N [yi×log⁡(pi)+(1-yi)×log⁡(1-pi)]

where log denotes the natural logarithm.

## 3 Results

### 3.1 Comparison with deep learning baseline models

In this section, we evaluate the performance of 2OMe-LM by comparing it against several deep learning baseline models using five-fold cross-validation. The training dataset was evenly partitioned into five subsets. In each fold, one subset (20%) served as the validation set, while the remaining four subsets (80%) were used for training. This process was repeated final performance was determined by averaging the results across all five-folds. Table 1 presents the performance of 2OMe-LM and the deep learning baseline models. From Table 1, we can observe that 2OMe-LM outperforms all deep learning baseline models across various evaluation metrics. The results demonstrate the superior capability of 2OMe-LM’s network architecture in accurately identifying 2OMe sites.

### 3.2 Comparison with existing state-of-the-art predictors

**Figure 2.**
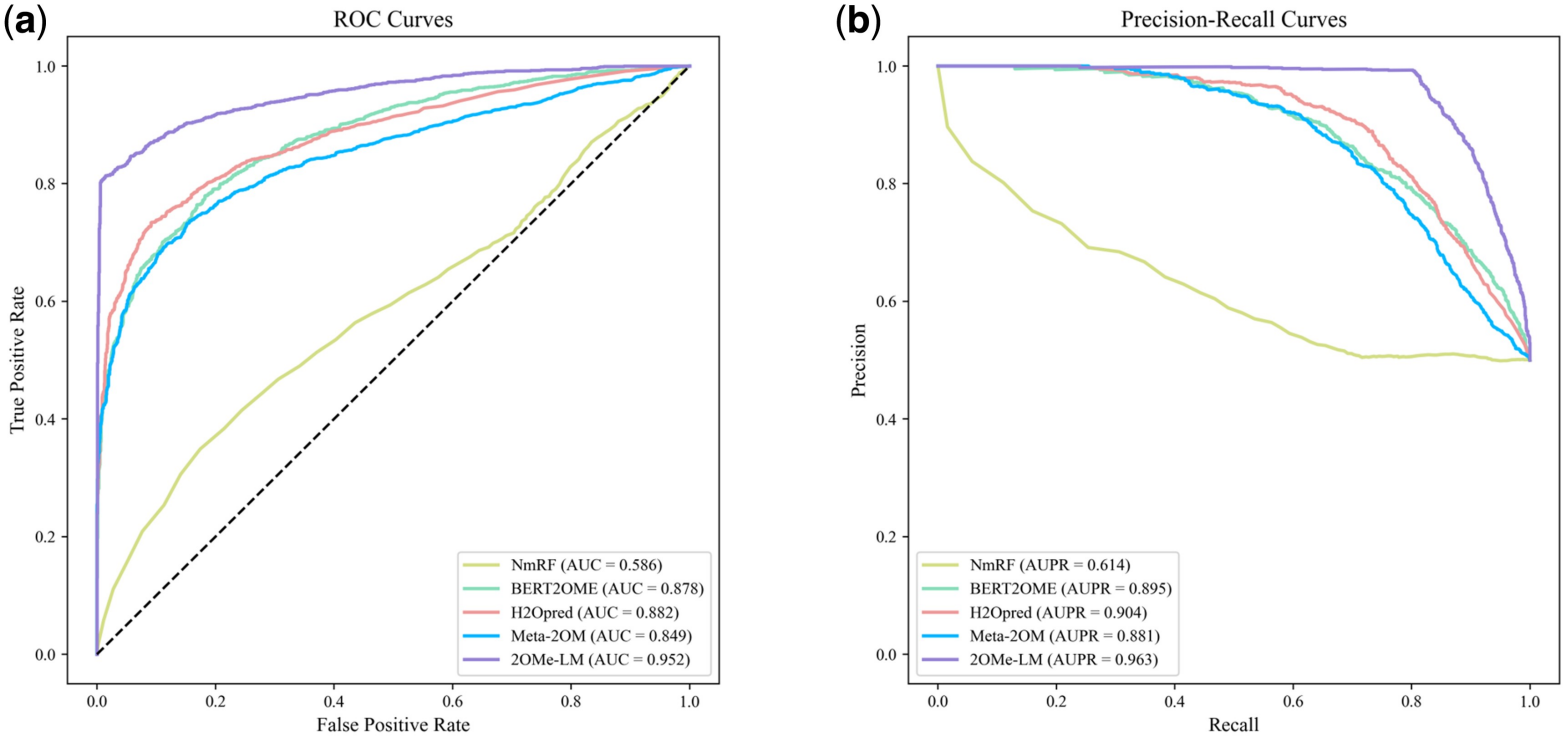
The ROC and PR curves of 2OMe-LM and existing predictors on the test set. (a) The ROC curves. (b) The PR curves.

To evaluate the performance of 2OMe-LM, we compared it with several existing computational methods. Given that most of these computational methods do not provide source code, we used their web server versions for comparison. The selection criteria for current predictors included: (i) availability of a web server or stand-alone version; (ii) input that only requires RNA sequences; and (iii) outputs that include predictive scores. Based on these criteria, we identified four predictors: NmRF, BERT2OME, H2Opred and Meta-2OM. We used the web servers of NmRF (http://lab.malab.cn/∼acy/NmRF), H2Opred (https://balalab-skku.org/H2Opred/) and Meta-2OM (http://kurata35.bio.kyutech.ac.jp/Meta-2OM) for the evaluation. For BERT2OME, we trained the model on our benchmark dataset using its publicly available code repository (https://github.com/seferlab/bert2ome). Table 2 presents the performance of 2OMe-LM with existing predictors on the independent test set. 2OMe-LM exhibits superior performance, achieving ACC of 0.898, F1-score of 0.887, precision of 0.993, recall of 0.802, AUC of 0.952, AUPR of 0.963 and MCC of 0.811. Notably, 2OMe-LM outperforms all existing predictors across all evaluation metrics, except for the recall of NmRF. Figure 2 illustrates the ROC and PR curves of 2OMe-LM and the other predictors. As shown, 2OMe-LM achieves an AUC of 0.952 and an AUPR of 0.963, significantly surpassing the performance of the other predictors. In summary, these results clearly indicate that 2OMe-LM outperforms existing predictors in the prediction of 2OMe sites.

**Table 2.** Performance comparison of 2OMe-LM with existing predictors on the independent test set.^a^

Predictor	ACC	F1-score	Precision	Recall	AUC	AUPR	MCC
NmRF	0.512	0.624	0.508	**0.810**	0.586	0.614	0.030
BERT2OME	0.795	0.788	0.813	0.765	0.878	0.895	0.591
H2Opred	0.819	0.800	0.890	0.727	0.882	0.904	0.648
Meta-2OM	0.780	0.744	0.891	0.639	0.849	0.881	0.584
2OMe-LM	**0.898**	**0.887**	**0.993**	0.802	**0.952**	**0.963**	**0.811**

a The best performance values are highlighted in bold.

### 3.3 Effectiveness of pre-trained RNA language model

In the study, we used a pre-trained RNA language model, SpliceBERT, to encode RNA sequences. To evaluate the contribution of the pre-trained RNA language models. We conducted some analyses.

First, we performed an ablation study by constructing two variant models of 2OMe-LM: 2OMe-LM-exclude-LM and 2OMe-LM-exclude-word2vec. The former removes SpliceBERT from 2OMe-LM, while the latter excludes the word2vec component. The five-fold cross-validation performance of these models on the entire dataset is presented in Table 3. The results of the ablation study reveal that both feature representation methods enhance the performance of 2OMe-LM, with the removal of either method leading to a noticeable decline in performance. Importantly, SpliceBERT have a more pronounced impact on the overall performance of 2OMe-LM. The results confirm that the embedding vectors generated by SpliceBERT are more effective than those generated by the word2vec technique.

**Table 3.** The performance of 2OMe-LM and its variant models in the ablation study.^a^

Model	ACC	F1-score	Precision	Recall	AUC	AUPR	MCC
2OMe-LM-exclude-LM	0.819	0.821	0.815	**0.829**	0.902	0.913	0.640
2OMe-LM-exclude-word2vec	0.833	0.830	0.846	0.815	0.910	0.922	0.666
2OMe-LM	**0.846**	**0.841**	**0.873**	0.811	**0.919**	**0.929**	**0.695**

a The best performance values are highlighted in bold.

**Figure 3.**
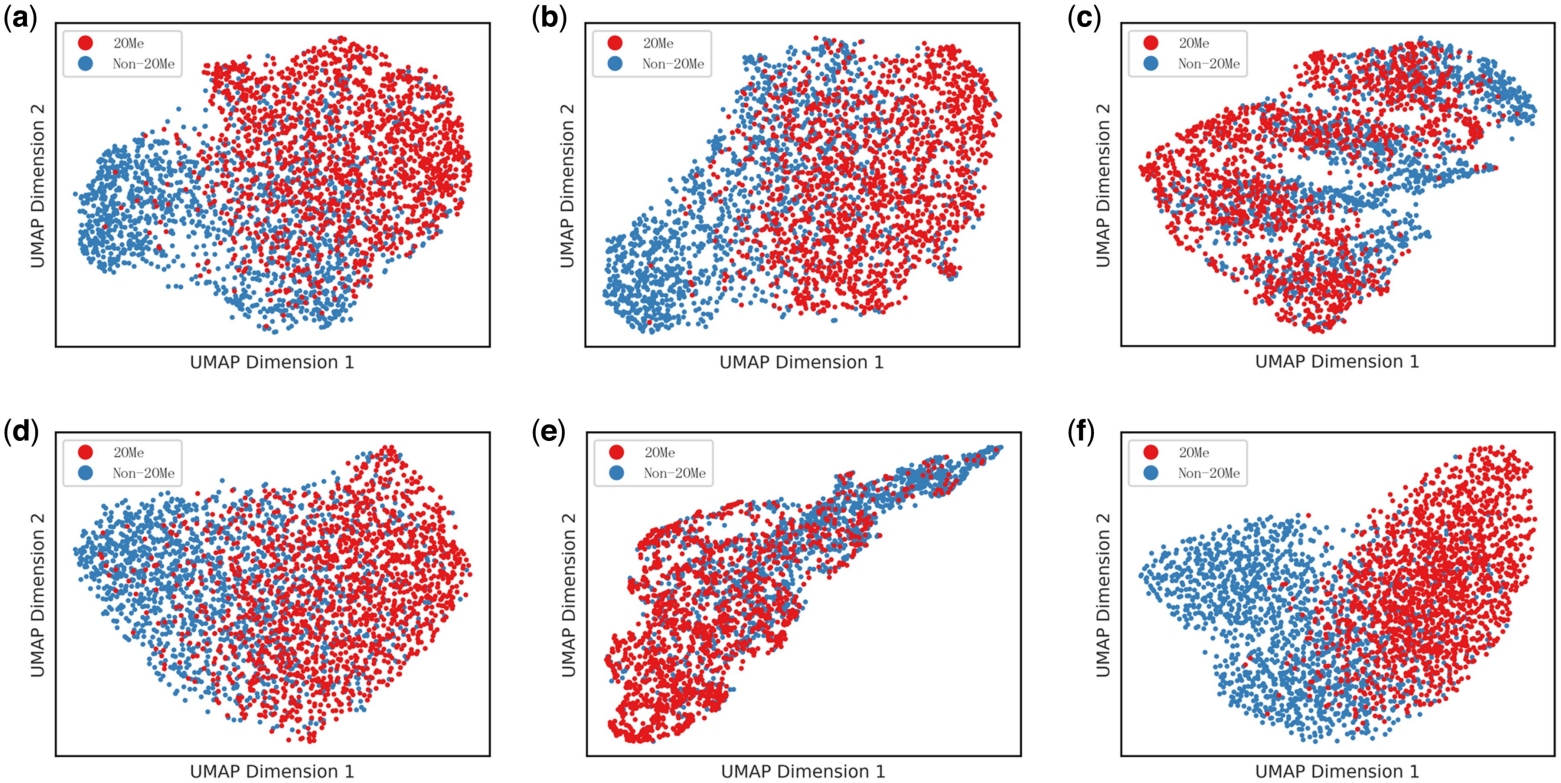
UMAP visualization results of using different encoding methods. Each dot represents a sample and its color represents its true class. Each subplot represents the UMAP visualization of the embedding vectors obtained from (a) using one-hot coding, (b) using ENAC features, (c) using ANF features, (d) using PseDNC features, (e) using PCP features and (f) using features derived from SpliceBERT.

To further illustrate the performance of the pre-trained RNA language model, we compared it with several commonly used RNA sequence representation methods, including one-hot coding, accumulated nucleotide frequency (ANF), enhanced nucleic acid composition (ENAC), pseudo-dinucleotide composition (PseDNC) and physical–chemical properties (PCP). A detailed description of these RNA sequence representation methods is provided in Text 1, available as supplementary data at *Bioinformatics* online. We followed the methodology outlined by Chari and Pachter (2023), visualized the embedding vectors by projecting them into a two-dimensional space using uniform manifold approximation and projection (UMAP) (McInnes *et al.* 2018), as shown in Fig. 3. In Fig. 3, red dots represent 2OMe site samples, while blue dots represent non-2OMe site samples. Figure 3a–e displays the UMAP visualization results of using one-hot coding, ENAC features, ANF features, PseDNC features and PCP features, respectively. Figure 3f illustrates the UMAP visualization results of using features derived from SpliceBERT. Notably, Fig. 3f demonstrates a clear distinction between 2OMe site samples and non-2OMe site samples, indicating that the pre-trained RNA language model generates more distinguishable embedding vectors for 2OMe site and non-2OMe site samples.

### 3.4 Motif analysis

**Figure 4.**
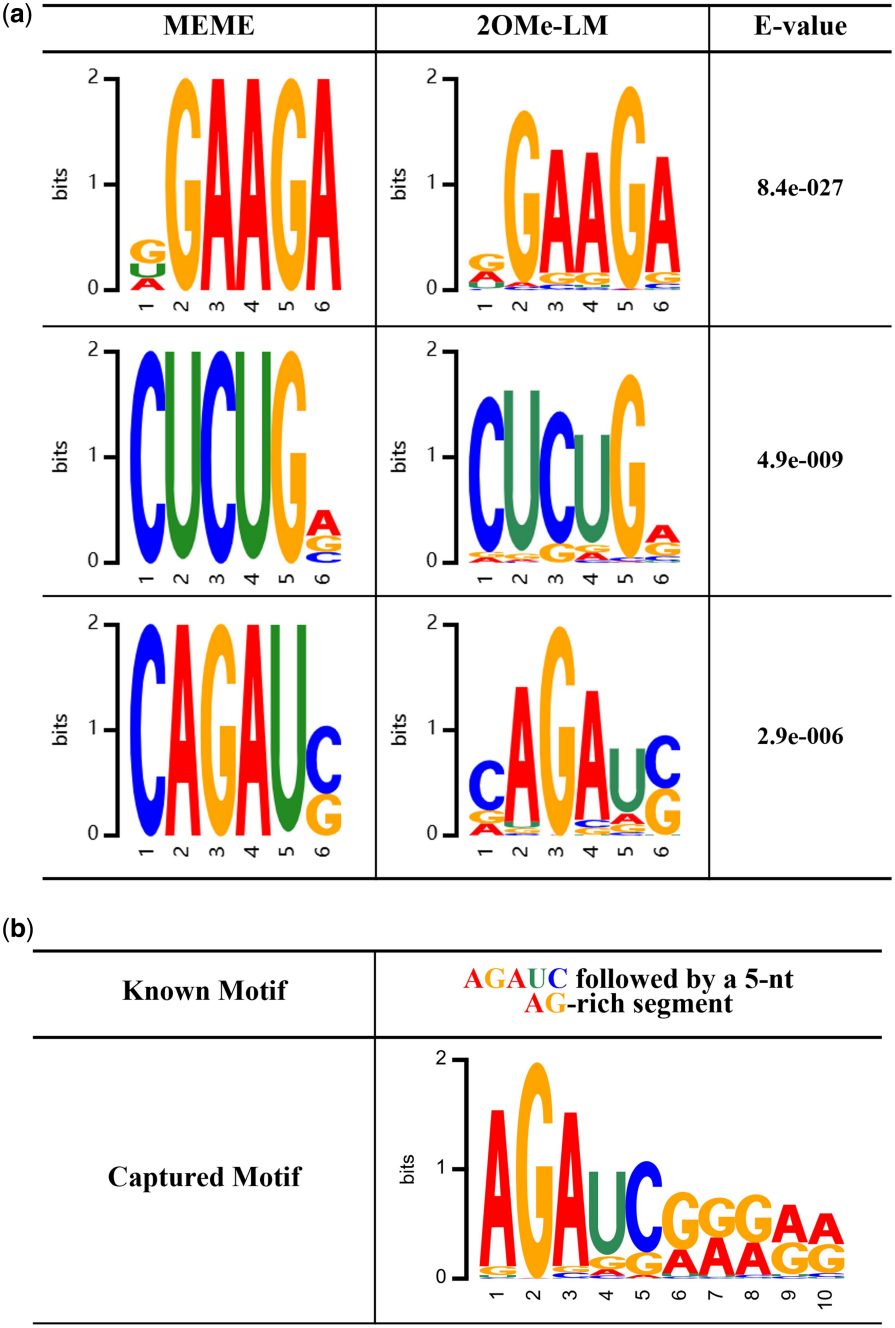
(a) Motifs discovered by MEME suite (left) and by 2OMe-LM (middle). The right are the *E*-values of the motifs found by the MEME suite. (b) 2OMe-LM captures a known 2OMe motif. The known motif is AGAUC followed by a 5-nt AG-rich segment.

In 2OMe-LM, we employed a multi-head self-attention mechanism to capture the attention weights of each nucleotide within RNA sequences. To evaluate the effectiveness of the mechanism in 2OMe-LM, we conducted a comprehensive motif analysis. First, we tested whether 2OMe-LM could identify the most frequently occurring motifs. Specifically, we used the MEME suite (Bailey *et al.* 2009) to identify motifs in our dataset of all 2OMe site samples, focusing on motifs with a width of 6 and setting the *E*-value threshold to 0.05. For 2OMe-LM, we calculated the attention weight of each nucleotide across RNA sequences, using the mean weight per sequence as a threshold to determine significant attention. A nucleotide is considered significant if its attention weight exceeded this threshold, indicating that 2OMe-LM pays attention to it. Conversely, nucleotides with attention weights below the threshold are regarded as ignored by 2OMe-LM. Figure 4a presents representative examples: the left column displays motifs discovered by MEME, the centre column illustrates motifs captured by 2OMe-LM, and the right column shows the *E*-values of motifs identified by MEME. As shown in Fig. 4a, 2OMe-LM successfully captures motifs similar to those identified by MEME, indicating that 2OMe-LM can capture frequently repeated motifs.

Furthermore, we investigated whether 2OMe-LM could identify known motifs associated with 2OMe sites. Specifically, we searched recent literature for experimentally verified motifs related to 2OMe modification. Dai *et al.* (2017) identified a signature sequence motif for 2OMe, which consists of the AGAUC motif followed by a 5-nt AG-rich segment. We used this motif as a case study to demonstrate the performance of 2OMe-LM, with the motifs captured by 2OMe-LM illustrated in Fig. 4b. As shown in Fig. 4b, 2OMe-LM successfully capture motifs that closely resemble known motifs, highlighting its capability to detect biologically relevant patterns.

### 3.5 Case study

**Figure 5.**
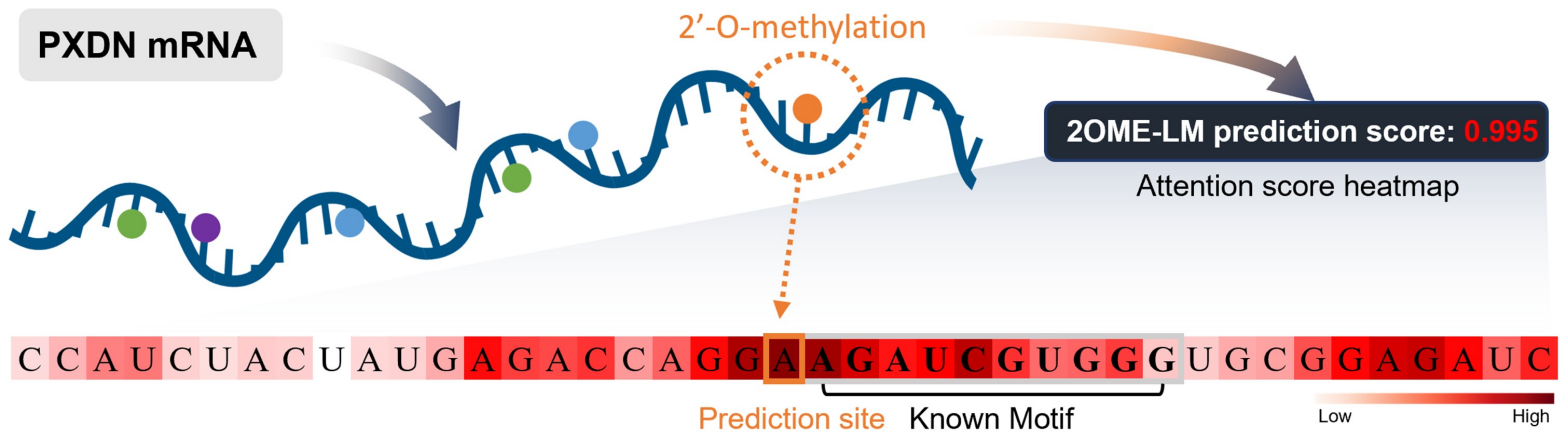
Prediction results of the 2OMe-site in PXDN mRNA using 2OMe-LM. 2OMe-LM successfully predicts the 2OMe site with a predicted probability of 0.995. The accompanying nucleotide weight heatmap demonstrates that 2OMe-LM captures the hallmark motif of 2OMe, specifically comprising the AGAUC motif followed by a 5-nt segment rich in AG.

To better understand the mechanism of 2OMe-LM, we conducted a case study using peroxidasin (PXDN) mRNA (NCBI Reference Sequence: NM_012293.3). Gumienny *et al.* (2017) confirmed the presence of 2OMe on this mRNA using RiboMeth-seq (GEO Accession: GSE77024). Previous studies (Elliott *et al.* 2019) indicated that this 2OMe modification enhances PXDN mRNA expression while inhibiting its translation, thereby regulating PXDN protein expression and enzymatic activity both *in vitro* and *in vivo*. We identified the specific position of the 2OMe modification in PXDN mRNA using data from the GEO (Accession: GSE77024). Then, we extracted a 41-nt segment consisting of 20-nt upstream and 20-nt downstream, as input for 2OMe-LM to perform predictions. The results are shown in Fig. 5. From Fig. 5, we can observe that 2OMe-LM successfully predicted the site as a 2OMe site, with a predicted probability of 0.995. The accompanying nucleotide weight heatmap illustrates the attention weights, with deeper red regions indicating greater weight and lighter regions indicating lesser weight. The visualization clearly demonstrates that 2OMe-LM effectively captures the hallmark motif of 2OMe identified by Dai *et al.* (2017) specifically comprising the AGAUC motif followed by a 5-nt segment rich in AG. The results align with their conclusion that such motifs typically appear downstream of the modification site.

### 3.6 Cross-nucleotide evaluation

**Figure 6.**
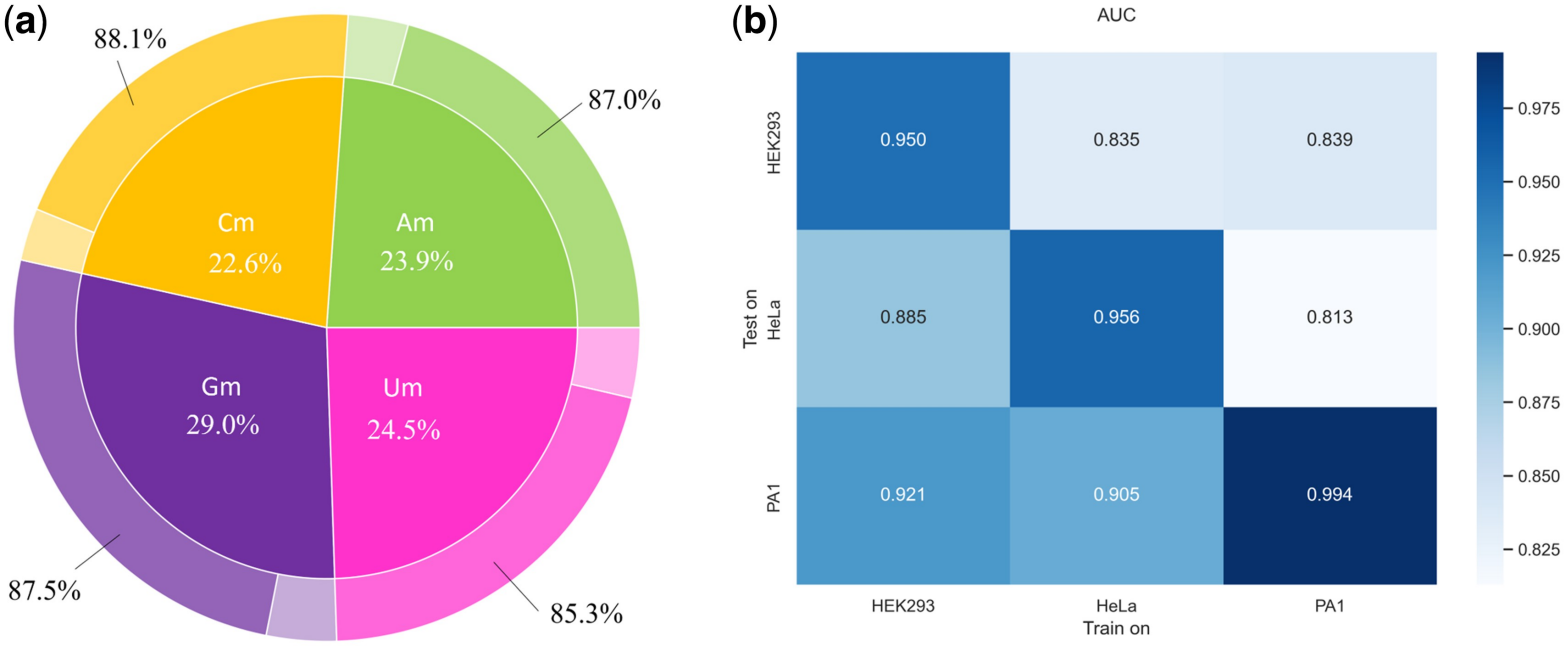
(a) Distribution of the four types of modifications (inner circle of the pie chart) and the accuracy of the 2OMe-LM model in predicting these modifications (outer circle of the pie chart). (b) The heatmap of AUCs for the cross-cell line evaluation. The horizontal axis represents the cell line used for model training, and the vertical axis represents the cell line used for evaluation.

Unlike modifications such as m6A and m5C, which occur directly on nucleotides, 2OMe takes place on the 2′-hydroxyl group of the RNA ribose. This allows 2OMe to be categorized into four types based on the nucleotide: Am, Cm, Gm and Um. To evaluate the performance of the 2OMe-LM model in identifying these four types of modifications, we divided the 2OMe site sample data into four subsets and performed predictions for each category separately. The results are shown in Fig. 6a. From Fig. 6a, we observe that high-confidence 2OMe modifications are most prevalent on uracil (U), followed by adenine (A) and cytosine (C). The lowest frequency is observed on guanine (G). Figure 6a also illustrates the accuracy of the 2OMe-LM model in predicting the four types of modifications. Overall, the model performs well, with Cm achieving the highest accuracy of 88.1%, followed by Gm at 87.5%, and Am and Um at 87.0% and 85.3%, respectively. Although the distribution of modifications is imbalanced (ranging from 22.6% for Cm to 29.0% for Gm), the accuracy remains stable (from 85.3% for Um to 88.1% for Cm). This suggests that the 2OMe-LM model is not affected by the imbalanced distribution of the four types of modifications and is capable of learning distinct patterns for each type. Other evaluation metrics of 2OMe-LM across different modification types in the independent test set are provided in Table 2, available as supplementary data at *Bioinformatics* online.

### 3.7 Cross-cell line evaluation

To explore the relationship of 2OMe sites across different cell lines and evaluate the model’s performance across cell lines, we collected datasets from three distinct cell lines: HEK293, HeLa and PA1, sourced from RMBase v3 (Table 3, available as supplementary data at *Bioinformatics* online). Specifically, we trained individual models on each cell line’s training set and evaluated their performance on the test sets of the other two cell lines. Figure 6b presents a heatmap showing the AUC values obtained during the cross-cell line evaluation. From Fig. 6b, we observe some interesting results: (i) The diagonal results are the highest, indicating that training and testing on the same cell line yields the best performance. (ii) There are significant differences in the results on the non-diagonal comparisons. Taking the Hela cell line as an example, when the model was trained on the Hela cell line and tested on the PA1 cell line, the AUC was 0.905. In contrast, when the model was trained on the Hela cell line and tested on the HEK293 cell line, the AUC was 0.835. This variation demonstrates that the environments of different cell lines are quite distinct, leading to substantial differences in performance. In addition, the other cross-cell line evaluation metrics shown in Fig. 1, available as supplementary data at *Bioinformatics* online, exhibit similar trends.

### 3.8 Model robustness evaluation

To evaluate the robustness of 2OMe-LM, we employed the fast gradient method (FGM) (Goodfellow *et al.* 2014), a straightforward and efficient adversarial attack technique. FGM generates adversarial samples by adding small, gradient-based perturbations to the model’s input, thereby inducing precise disturbances that can mislead the model’s predictions. A detailed description of the FGM method is provided in Text 2, available as supplementary data at *Bioinformatics* online. First, we tested the trained model on the human independent test set with adversarial samples generated via FGM. The results are shown in Table 4. The variations of evaluation metrics between the original and adversarial samples show that all changes remain within 5%, suggesting only slight changes in performance under adversarial perturbations. These results suggest that the performance of 2OMe-LM is minimally affected by FGM-induced perturbations, demonstrating its robustness. One possible reason for the phenomena is the feature encoding derived from SpliceBERT, which has been pre-trained on a large corpus of RNA sequences. This provides the model with rich sequence pattern recognition capabilities, allowing it to maintain prediction performance under minor perturbations or adversarial attacks.

**Table 4.** Performance of 2OMe-LM on original and adversarial samples on the test set.^a^

	ACC	F1-sorce	Precision	Recall	AUC	AUPR	MCC
Original samples	0.898	0.887	0.993	0.802	0.952	0.963	0.811
Adversarial samples	0.882	0.868	0.977	0.782	0.934	0.966	0.785
Change	−1.78%	−2.14%	−1.61%	−2.49%	−1.89%	+0.31%	−3.21%

a Use + or − symbols to indicate whether each metric has increased or decreased.

### 3.9 Web server

To facilitate the use of 2OMe-LM, we developed a user-friendly web server, accessible at https://csuligroup.com:9200/2OMe-LM. The 2OMe-LM web server requires RNA sequences of 41 nt as input. Users can upload sequence files in FASTA format, or directly enter one or more RNA sequences in FASTA format in the input box. After entering the sequences, users can click the submit button to run the prediction task. Once the prediction task is complete, the prediction results will be displayed on the web page, and users can download the prediction results in csv format for further analysis.

## 4 Conclusion

2OMe is a critical RNA modification that plays an essential role in regulating gene expression and maintaining cellular homeostasis. In recent years, RNA language models have shown promising potential in RNA bioinformatics tasks. Inspired by the success of RNA language models, in the study, we propose a novel deep learning model, 2OMe-LM, which leverages a pre-trained RNA language model to predict 2OMe sites. 2OMe-LM combines features encoded by SpliceBERT and those encoded by the word2vec technique. These features are processed individually by a fully connected layer and a Bi-LSTM block before being fused together. 2OMe-LM uses a multi-head self-attention mechanism to assign attention weights to each nucleotide in the RNA sequence. Finally, the fused features pass through three fully connected layers to perform the final prediction. The results of an independent test set confirm the superiority of 2OMe-LM, outperforming existing state-of-the-art predictors. Notably, 2OMe-LM is the first model to use a pre-trained RNA language model for 2OMe site prediction, enhancing both robustness and generalizability.

Despite the promising results of 2OMe-LM, certain limitations remain for improvement. In particular, the model fails to achieve satisfactory performance when applied to cross-species prediction tasks. We attribute this limitation to three primary factors: (i) The scarcity of data from other species makes it difficult to evaluate the model effectively, the results obtained from limited datasets may not accurate reflect real-world scenarios. (ii) Certain species may possess unique sequence features, RNA-modifying enzymes, cofactors, or regulatory networks, which can lead to substantially different 2OMe patterns compared to those observed in the species used for model training. (iii) Differences in RNA secondary and tertiary structures across species can significantly affect the accessibility and efficiency of modification sites. These structural variations are difficult for sequence-based models to capture accurately. In the future, if we can collect more reliable cross-species datasets, it could enhance the model’s cross-species prediction performance. In addition, we plan to integrate multimodal features, including RNA structural and functional information along with other omics data, to develop multimodal learning framework to optimize model performance for cross-species 2OMe site prediction. Furthermore, we aim to explore more advanced representation learning techniques, such as transforming RNA sequences into graph structures and employing contrastive learning strategies, to enhance 2OMe modification site prediction.

## Supplementary Material

btaf417_Supplementary_Data

## Data Availability

All data used in this study are available at https://github.com/CSUBioGroup/2OMe-LM.
