# Supplementary material for: 2OMe-LM: predicting 2′-O-methylation sites in human RNA using a pre-trained RNA language model
Source: Bioinformatics. 2025 Jul 29;41(8):btaf417. doi: 10.1093/bioinformatics/btaf417 (PMC12342186; doi:10.1093/bioinformatics/btaf417)
Supplement: btaf417_Supplementary_Data [file btaf417_supplementary_data.pdf]

Supplementary materials:

2OMe-LM: predicting 2’-O-methylation sites in human RNA using a pretrained RNA language model

Qianpei Liu[1, #], Min Zeng[1, #], Yiming Li[1], Chengqian Lu[2], Shichao Kan[1], Fei Guo[1], and Min Li[1, *]

[1]School of Computer Science and Engineering, Central South University, Changsha, 410083, China

[2]School of Computer Science, Key Laboratory of Intelligent Computing and Information Processing, Xiangtan University, Xiangtan, Hunan, 411105, China

[#] These authors contributed equally to this work

* Correspondence to: Min Li, E-mail: limin@mail.csu.edu.cn

The supplementary files include:

1. Supplementary Texts

Text S1. Traditional encoding methods for RNA sequences.
Text S2. Fast Gradient Sign Method.

2. Supplementary Figures

Figure S1. The heatmap of performances for the cross-cell line evaluation. The darker blue colors indicate the higher AUC values. The horizontal axis represents the cell line used for model training, and the vertical axis represents the cell line used for evaluation. (a) ACC, (b) F1-sore, (c) Precision, (d) Recall, (e) AUPR, (f) MCC.

3. Supplementary Tables

Table S1. A statistical summary of the datasets used in the study.
Table S2. Performance of four types of modifications 2OMe-LM on the independent test set.
Table S3. A statistical summary of the datasets used in the cross-cell line evaluation.

1. Supplementary Texts

Text S1. Traditional encoding methods for RNA sequences.

One-hot： This method converts RNA sequences into a numerical format that can be processed by computational models, crucial for subsequent biological data analysis and the training of machine learning models. The principle of this encoding is to map each nucleotide (A, U, C, G) to a unique binary vector. For example, A is encoded as [1, 0, 0, 0], U as [0, 1, 0, 0], C as [0, 0, 1, 0], and G as [0, 0, 0, 1]. Each vector has a fixed length, with only one position marked as 1 and the rest as 0, reflecting the type of nucleotide at that position. Through one-hot encoding, an RNA sequence with a length of L nucleotides are encoded into a $L \times 4$ binary vector.

Enhanced Nucleotide Composition (ENAC)： In this approach, a sequence is divided into overlapping k-mers (short nucleotide fragments of length k), and the frequency of each unique k-mer across the sequence is calculated. The frequency of a k-mer $k - mer_i$ in an RNA sequence can be calculated with the following formula:

$$Frequency(k - mer_i) = \frac{Count(k - mer_i)}{L - k + 1} \tag{1}$$

where $k - mer_i$ is a specific nucleotide fragment of length k in the RNA sequence, $Count(k - mer_i)$ is the number of times $k - mer_i$ appears in the sequence and L is the total length of the RNA sequence. Each k-mer's frequency is stored as a numerical value, creating a vector of length 4^k, where each element represents the occurrence frequency of a specific k-mer in the RNA sequence. By capturing k-mer frequencies, ENAC encoding provides a more comprehensive representation of the sequence's structural composition, making it beneficial for certain biological data analyses and machine learning models that can leverage k-mer patterns for sequence classification tasks. This approach is particularly advantageous in identifying subtle patterns related to functional or structural properties of RNA sequences.

Accumulated Nucleotide Frequency (ANF): This approach aims to capture the positional distribution and composition of nucleotides (A, U, C, G) across the entire sequence, providing a richer representation of the sequence's nucleotide pattern than basic frequency counts. In ANF encoding, for each position i in the sequence, the cumulative frequency of a nucleotide x is calculated up to that position. The cumulative frequency of nucleotide x at position i is given by:

$$ANF(x, i) = \frac{Count(x, i)}{i} \tag{2}$$

where $Count(x, i)$ is the number of times nucleotide x has appeared from the start of the sequence up to position i, i is the current position within the sequence. Each nucleotide x has an ANF value calculated at each position i, creating a feature vector where each entry represents the cumulative frequency of a nucleotide up to that point in the sequence. This encoding results in a vector of length $4 \times L$, where L is the total length of the sequence, with each nucleotide's cumulative frequency contributing to the overall encoding. By capturing the cumulative frequencies of nucleotides along the sequence, ANF encoding emphasizes the distribution patterns of nucleotides, making it useful for tasks where positional nucleotide accumulation is relevant. This can be particularly advantageous in machine learning models for sequence classification and analysis, as it allows the model to capture both nucleotide composition and distribution simultaneously.

Pseudo Dinucleotide Composition (PseDNC): PseDNC is a specific instance of Pseudo K-tuple Nucleotide Composition (PseKNC), where $k = 2$, meaning it focuses on capturing both the composition and sequence-order information of dinucleotides. PseKNC is a generalized framework used to represent nucleotide sequences by retaining global or long-range sequence-order information, combining k-tuple nucleotide composition with sequence-order correlation. When $k = 2$, this framework is called PseDNC, and it specifically focuses on encoding RNA sequences with dinucleotide information. The PseDNC encoding vector D_{PseKNC} is structured as follows:

$$D_{\text{PseKNC}} = \left[d_1, \cdots, d_{4^k}, d_{4^k+1}, \cdots, d_{4^k+\alpha}\right]^T (\alpha < L - k) \tag{3}$$

where L is the length of the RNA sequence, 4^k resents the number of possible k-tuple nucleotide combinations (for PseDNC, $k = 2$, so there are $4^2 = 16$ possible dinucleotide combinations),α is the number of pseudo components that capture sequence-order information.

Each component d_u in the encoding vector is computed as follows:

$$d_u = \begin{cases} \dfrac{f_u^k}{\sum_{i=1}^{4^k} f_i^k + \omega \sum_{j=1}^{\alpha} \theta_j} & (1 \le u < 4^k) \\ \dfrac{\omega \theta_{u-4^k}}{\sum_{i=1}^{4^k} f_i^k + \omega \sum_{j=1}^{\alpha} \theta_j} & (4^k + 1 \le u < 4^k + \alpha) \end{cases} \tag{4}$$

where f_u^k is the normalized frequency of the u-th dinucleotide in the RNA sequence, ω is a weight factor that balances the contributions of k-tuple nucleotide composition and sequence-order correlation, θ_j is the j-th order correlation factor, representing the long-range correlation between contiguous k-tuples and it is calculated as:

$$\theta_j = \frac{1}{L - j - 1} \sum_{i=1}^{L-j-1} C_{i,i+j} \tag{5}$$

The sequence-order correlation function $C_{i,i+j}$ measures the relationship between two dinucleotides at positions i and $i + j$, calculated as:

$$C_{i,i+j} = \frac{1}{\mu} \sum_{g=1}^{\mu} \left[P_{C_g}(D_i) - P_{C_g}(D_{i+j})\right]^2 \tag{6}$$

where μ is the number of physicochemical properties considered, $P_{C_g}(D_i)$ is the value of the g-th physicochemical property for the dinucleotide D_i at position i in the RNA sequence.

Physical-Chemical Properties (PCP): PCP of dinucleotides are essential features for generating PseKNC representations, as they capture various physicochemical characteristics of dinucleotide pairs within an RNA sequence. These properties help retain sequence-order information by providing insights into the structural and energetic attributes of nucleotides and their interactions. In the PseKNC framework, PCP values of dinucleotides can serve as feature components, helping models learn not only from nucleotide composition but also from specific molecular properties. The following 10 physical-chemical properties are often considered for encoding dinucleotides in PseKNC:

1) Rise – Describes the vertical distance between successive base pairs.
2) Roll – Measures the tilt between successive base pairs.
3) Shift – Reflects the horizontal displacement between bases.
4) Slide – Represents the sliding motion of one base pair relative to another.

5) Tilt – Shows the inclination angle of bases.
6) Twist – Indicates the helical twist between base pairs.
7) Enthalpy – Represents the heat content or stability of base pairs.
8) Entropy – Describes the disorder associated with the base pair.
9) Stack Energy – Measures the stacking interaction energy between base pairs.
10) Free Energy – Refers to the overall energy that influences molecular stability.

For a given RNA sequence, the physical-chemical property matrix PC can be defined to encapsulate these attributes across all dinucleotides. This matrix is structured as follows:

$$PC = \begin{bmatrix} PC^1(N_1N_2) & PC^1(N_2N_3) & \dots & PC^1(N_{L-1}N_L) \\ PC^2(N_1N_2) & PC^2(N_2N_3) & \dots & PC^2(N_{L-1}N_L) \\ \vdots & \vdots & \ddots & \vdots \\ PC^{10}(N_1N_2) & PC^{10}(N_2N_3) & \dots & PC^{10}(N_{L-1}N_L) \end{bmatrix} \quad (7)$$

where $PC^i(N_jN_{j+1})$ represents the i-th physicochemical property value for the dinucleotide at the j-th position in the sequence, L is the length of the RNA sequence, so the matrix has dimensions $10 \times (L-1)$, covering all dinucleotide pairs across all 10 properties. To ensure consistency and comparability, the values of each physical-chemical property must undergo normalization. This step standardizes the properties, making them suitable for use in computational models and reducing potential biases due to scale differences among properties.

Text S2. Fast Gradient Sign Method.

The Fast Gradient Sign Method (FGM) is a commonly used adversarial attack technique designed to assess the robustness of machine learning models. By introducing controlled perturbations to the input, FGM can test a model's vulnerability to adversarial examples, which aim to mislead the model into incorrect classifications. The FGM approach works by calculating a perturbation, δ, that maximizes the model's loss function. The perturbation δ is defined as:

$$\delta = \epsilon \cdot sign\big(\nabla_x J(\theta, x, y)\big) \quad (8)$$

where x denotes the original input vector, y is the corresponding true label vector, J is the loss function, typically cross-entropy in classification tasks, ϵ is a constant controlling the perturbation magnitude, which is often referred to as the "attack budget." The gradient $\nabla_x J(\theta, x, y)$ represents the direction in which the input should be perturbed to increase the loss. The function $sign(\cdot)$ ensures that each element of the perturbation vector δ points in the positive or negative direction that maximizes the loss function, effectively creating a directional “push” to mislead the model.

Once δ is calculated, it is applied to the original input x to generate the adversarial example x_{attack}:

$$x_{attack} = x + \delta \quad (9)$$

This adversarial sample x_{attack} simulates an input with slight modifications intended to fool the model. By evaluating the model's performance on these adversarial samples, FGM provides insight into the model's robustness and helps identify potential vulnerabilities in handling noisy or perturbed RNA sequence data.

2. Supplementary Figures

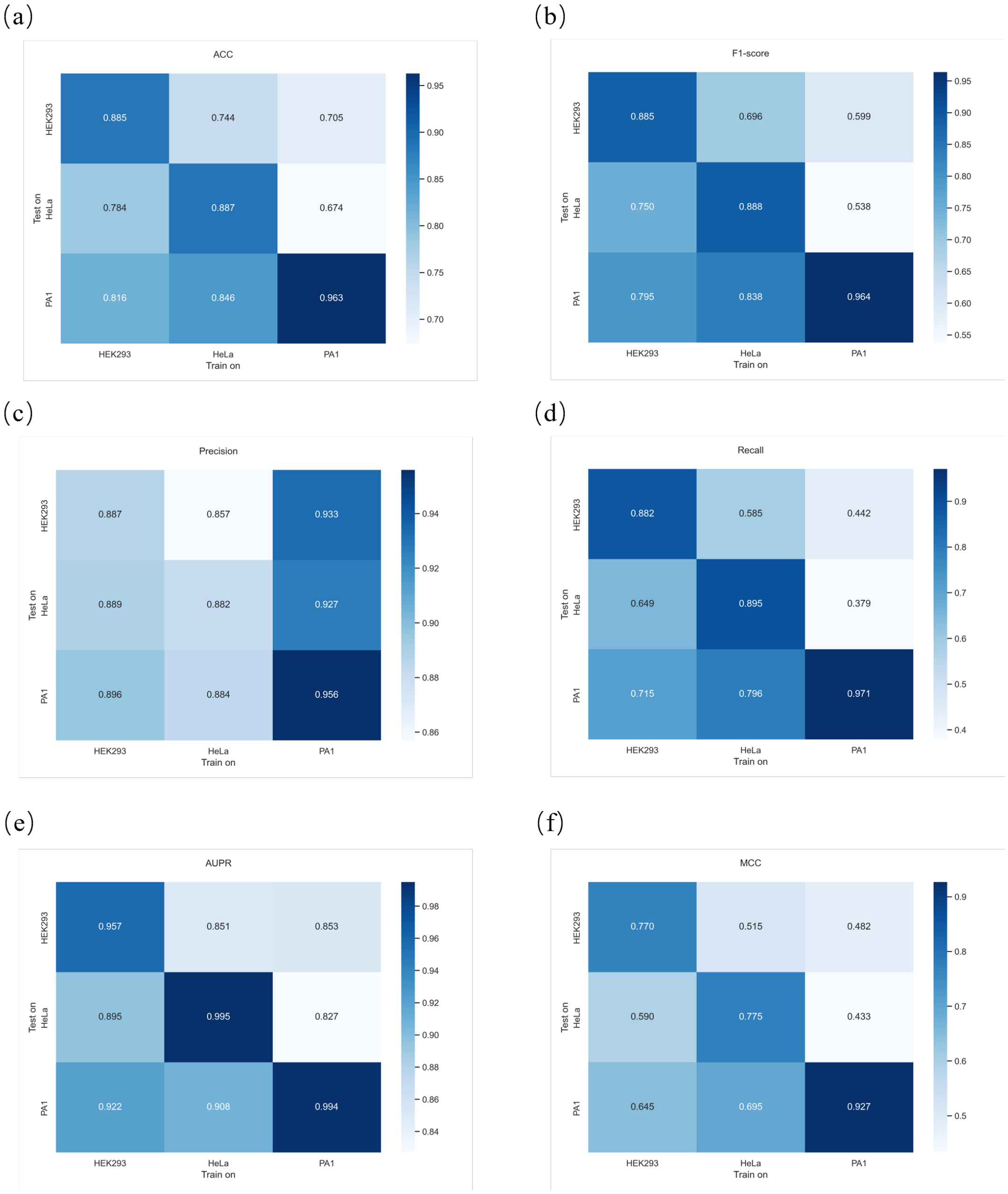

Figure S1. The heatmap of performances for the cross-cell line evaluation. The darker blue colors indicate the higher AUC values. The horizontal axis represents the cell line used for model training, and the vertical axis represents the cell line used for evaluation. (a) ACC, (b) F1-sore, (c) Precision, (d) Recall, (e) AUPR, (f) MCC.

3. Supplementary Tables

Table S1. A statistical summary of the datasets used in the study.

Datasets	# of 2OMe sites	# of non-2OMe sites
Training set	6429	6429
Independent set	1608	1608

Table S2. Performance of four types of modifications 2OMe-LM on the independent test set.

Type	ACC	F1-score	Precision	Recall	AUC	AUPR	MCC
Am	0.910	0.893	1.000	0.806	0.956	0.962	0.831
Cm	0.896	0.886	0.993	0.800	0.951	0.963	0.808
Um	0.872	0.856	0.988	0.755	0.937	0.948	0.766
Gm	0.914	0.911	0.992	0.842	0.964	0.974	0.839

Table S3. A statistical summary of the datasets used in the cross-cell line evaluation.

Datasets	# of 2OMe sites	# of non-2OMe sites
HEK293	1098	1098
HeLa	1694	1694
PA1	121	121
